# An engineered micropattern to reduce bacterial colonization, platelet adhesion and fibrin sheath formation for improved biocompatibility of central venous catheters

**DOI:** 10.1186/s40169-015-0050-9

**Published:** 2015-02-26

**Authors:** Rhea M May, Chelsea M Magin, Ethan E Mann, Michael C Drinker, John C Fraser, Christopher A Siedlecki, Anthony B Brennan, Shravanthi T Reddy

**Affiliations:** Sharklet Technologies, Inc, 12635 E. Montview Blvd. Suite 155, Aurora, CO 80045, CO USA; Departments of Bioengineering and Surgery, Pennsylvania State University, Hershey, PA USA; Departments of Materials Science and Engineering and Biomedical Engineering University of Florida, Gainesville, FL 32611 USA

**Keywords:** Sharklet, Microtopography, Platelet activation, Blood compatibility, Infection, CRT, CRBSI

## Abstract

**Background:**

Catheter-related bloodstream infections (CRBSIs) and catheter-related thrombosis (CRT) are common complications of central venous catheters (CVC), which are used to monitor patient health and deliver medications. CVCs are subject to protein adsorption and platelet adhesion as well as colonization by the natural skin flora (*i.e. Staphylococcus aureus* and *Staphylococcus epidermidis*). Antimicrobial and antithrombotic drugs can prevent infections and thrombosis-related complications, but have associated resistance and safety risks. Surface topographies have shown promise in limiting platelet and bacterial adhesion, so it was hypothesized that an engineered Sharklet micropattern, inspired by shark-skin, may provide a combined approach as it has wide reaching anti-fouling capabilities. To assess the feasibility for this micropattern to improve CVC-related healthcare outcomes, bacterial colonization and platelet interactions were analyzed *in vitro* on a material common for vascular access devices.

**Methods:**

To evaluate bacterial inhibition after simulated vascular exposure, micropatterned thermoplastic polyurethane surfaces were preconditioned with blood proteins *in vitro* then subjected to a bacterial challenge for 1 and 18 h. Platelet adhesion was assessed with fluorescent microscopy after incubation of the surfaces with platelet-rich plasma (PRP) supplemented with calcium. Platelet activation was further assessed by monitoring fibrin formation with fluorescent microscopy after exposure of the surfaces to platelet-rich plasma (PRP) supplemented with calcium in a flow-cell. Results are reported as percent reductions and significance is based on *t*-tests and ANOVA models of log reductions. All experiments were replicated at least three times.

**Results:**

Blood and serum conditioned micropatterned surfaces reduced 18 h *S. aureus* and *S. epidermidis* colonization by 70% (p ≤ 0.05) and 71% (p < 0.01), respectively, when compared to preconditioned unpatterned controls. Additionally, platelet adhesion and fibrin sheath formation were reduced by 86% and 80% (p < 0.05), respectively, on the micropattern, when compared to controls.

**Conclusions:**

The Sharklet micropattern, in a CVC-relevant thermoplastic polyurethane, significantly reduced bacterial colonization and relevant platelet interactions after simulated vascular exposure. These results suggest that the incorporation of the Sharklet micropattern on the surface of a CVC may inhibit the initial events that lead to CRBSI and CRT.

## Background

Approximately six million central venous catheters (CVCs) are inserted each year in the U.S. [1] to provide efficient vascular monitoring and medication delivery. Thermoplastic polyurethane (TPU) has been adopted as the material of choice for CVCs due to its workability, resiliency and improved blood compatibility over other plastics [2]. CVC design improvements, such as valves and cuffs, have been introduced to prevent tube occlusion and limit microbial access to the bloodstream. Unfortunately even with these improvements, CVCs lead to the downstream complications of catheter-related blood stream infections (CRBSIs) and thrombosis (CRT), which are associated with increased mortality [3,4].

CRBSIs are primarily caused by the patient’s skin flora (e.g. *Staphylococcus aureus, S. epidermidis,* Candida spp.) that colonize the device and enter the blood stream [5]. Education for personnel who insert catheters, use of sterile barrier precautions, and chlorhexidine skin antisepsis during insertion have helped to reduce, but not eliminate, CRBSI rates [1,6,7]. Antimicrobial catheters also show promise in reducing CRBSI rates [8]. However, studies show that these catheters harbor microorganisms [9] and this constant exposure contributes to the rise in antimicrobial resistance among pathogens [10,11].

Factors that lead to thrombus formation and subsequent CRT involve a complex series of events beginning with the deposition of a variety of blood proteins and tissue factors that together facilitate platelet adhesion to a surface [12,13]. Platelets then activate and expedite the coagulation cascade, frequently leading to fibrin sheath formation along the device within 24 h [14]. Routine anticoagulation prophylaxis, blood-thinning therapy, and flushing of catheter ports, typically with heparin, are the only methods currently used to prevent CRT. Unfortunately, these treatments have not demonstrated efficacy against morbidity and mortality [14,15] and can lead to thrombocytopenia, where low platelet counts in the blood can cause hemorrhaging or an increased risk of CRT [16,17].

Several studies indicate that infection and thrombosis are clinically linked where patients with CRBSI often acquire CRT, and *vice versa* [18-23]. Controlled *in vivo* and *in vitro* studies have demonstrated that fibrin sheath on catheters increase bacterial colonization and correlate with positive blood cultures [24-27]. Alternatively, many bacterial strains, in addition to coagulase-positive *S. aureus*, can initiate the coagulation cascade resulting in fibrin clot formation [28,29]. These studies suggest that bacterial attachment or fibrin sheath formation on the surface of CVCs could be an underlying mechanism behind this strong clinical correlation.

Incorporation of nano and micropatterns into CVC surfaces has been proposed to reduce CRBSI and CRT rates without antimicrobial or antithrombogenic coatings. Specifically, ordered pillar topographies, with dimensions of 300 nm to 1 μm, have been shown to reduce platelet adhesion and aggregation [30,31]. The Sharklet micropattern, either with recessed (−3SK2×2) or protruding (+3SK2×2) features on the same size scale (*i.e.* 2 μm to 16 μm) contains an ordered arrangement of features bio-inspired by the diamond-like structure of shark skin. Sharklet micropatterns have been shown to control the bioadhesion of a wide range of marine microorganisms, pathogenic bacteria and eukaryotic cells [32-37]. The aim of the present study is to expand our understanding of the performance of the Sharklet micropattern against common microorganisms that cause CRBSIs after blood protein exposure as well as platelet adhesion and activation that leads to fibrin sheath formation and CRT.

## Methods

### Sample fabrication

**Figure 1:**
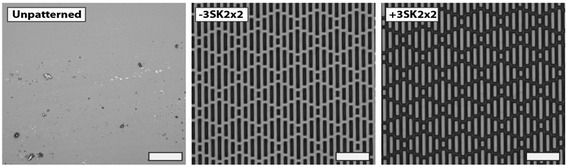
**Confocal microscopy images of a) unpatterned controls b)-3SK2x2 and c) +3SK2x2 Sharklet micropatterns replicated in TPU.** Representative images of the surfaces analyzed in this study. Scale bar, 20 μm.

Thermoplastic polyurethane (TPU) samples, with and without the Sharklet micropattern, were created by thermal embossing. Briefly, Tecoflex EG-85A pellets (Lubrizol) were heated and pressed in a Carver hydraulic press at 185°C and 40 MPa for 10 min to create a blank film. The resulting film was then embossed against a patterned or polished (smooth, unpatterned) nickel mold [34] to a thickness of ~0.4 mm in a Carver hydraulic press at 185°C and 40 MPa for an additional 2 min. Each flat 0.4 mm thick film was either punched into 12 mm diameter circular coupons or cut into 75 × 25 mm rectangles. The micropatterns produced by this technique were comprised of discontinuous channel features arranged in a Sharklet micropattern that either protruded (+) from or were recessed (−) into the polymer surface with features 3 μm tall or deep that were 2 μm wide and spaced by 2 μm; referred to throughout this study as +3SK2×2 and-3SK2×2 (Figure 1).

### Microbial strains, media, and growth conditions

Clinical isolates of *S. aureus* (ATCC 6538) and *S. epidermidis* (ATCC 35984) were analyzed in this study. A single colony of each bacterial strain, plated on tryptic soy agar (TSA; Criterion), was used to inoculate tryptic soy broth (TSB; Criterion) and grown in a shaking incubator at 37°C and 280 rpm overnight. Overnight microbial cultures were sub-cultured and grown to early log phase in TSB before centrifuging aliquots and resuspending cell pellets with 1× phosphate buffered saline (PBS; CulGeneX) to the desired inoculum concentration based on a previously-established ratio of optical density and CFU/ml.

### Staphylococcal colonization post blood protein pre-treatment

Bacterial aggregation, cell death, and clotting occurs when simultaneously incubating blood with *S. aureus* [38-42]. For *in vitro* studies, methods for surface pre-conditioning with blood or 25% serum, the physiologically relevant serum concentration found in blood, prior to bacterial inoculation were selected based on several published studies investigating the *in vitro* effects of blood-bacteria interactions for CVC applications [43-45]. Two agitation states were investigated as a CVC is subjected to a range of shear forces from static environments inside the lumen to flow rates of 17–81 ml/min in the blood vessel, depending on the tube size [46]. Micropatterned and unpatterned 12 mm TPU coupons (n=4) were pressed to the bottom of a sterile Petri dish around the outer perimeter. Coupons were immersed in either citrate-treated whole blood (Bonfils Blood Bank) or 25% serum in PBS (Interstate Blood Bank) for 2 h at 37°C either statically or dynamically (30 rpm) to match the inoculation condition. The pre-treatment media was rinsed three times by adding 20 ml of sterile PBS to the dish, swirling (80 rpm) for 30 s on an orbital shaker, and decanting the rinsate. Surfaces were then immersed in 20 ml of inoculum containing ~10^7^ (static) or ~10^8^ (dynamic) CFU/ml of either *S. aureus* or *S. epidermidis* and statically or dynamically incubated for 1 and 18 h at 37°C. After incubation, the inoculum suspension was decanted, surfaces were rinsed three times identically as washing following pre-treatment, punched with an 8 mm biopsy punch, vortexed and sonicated as described above. Bacterial eluate solutions were enumerated by a 10-fold dilution series, plating each dilution onto TSA, and counting the colony forming units (CFU) after sufficient growth at 37°C overnight. Each CFU per surface data point was log_10_ transformed before statistical analysis. Each experiment was replicated three times. Fluorescent microscopy was used to qualitatively verify the 18 h CFU results by fixing with 4% paraformaldehyde and staining with TOTO®-3. Images were obtained in three pre-selected sites per surface by confocal laser scanning microscopy (Zeiss LSM 510 META on Axiovert 200 M).

### Platelet adhesion

Micropatterned and unpatterned 12 mm TPU coupons (n=3) were pressed to the bottom of a sterile Petri dish around the outer perimeter. Surfaces were immersed in 20 ml of a final concentration of ~10^6^ platelets/μl derived from platelet rich plasma (Bonfils Blood Bank) diluted with PBS supplemented with 10 mM calcium chloride for 2 h 37°C while rotating at 80 rpm. Surfaces were rinsed three times by adding PBS to the dish, rotating (80 rpm) for 30 s on an orbital shaker, and decanting the rinsate. Platelets were fixed on surfaces for 1 h in 1% paraformaldehyde at room temperature. Fixed surfaces were blocked with 10% normal goat serum (Invitrogen) for 1 h before applying a primary α_IIb_β_3_ integrin antibody (ab662, abcam) diluted 1:1000 in 10% normal goat serum (Invitrogen) and incubating overnight at 4°C. Surfaces were exchange rinsed with PBS five times before adding secondary Alexa-Fluor-555 IgG antibody (Invitrogen) diluted 1:100 in 6% normal goat serum for 1 h at room temperature. Images were taken in 10 pre-determined locations per sample by fluorescent microscopy (Zeiss LSM 510 META on Axiovert 200 M), area coverage (μm^2^) was measured using ImageJ, and each coverage data point was log transformed prior to statistical analysis. Each experiment was replicated four times.

### Fibrin sheath formation

Micropatterned and unpatterned 75 × 25 mm TPU samples were loaded into a two-chamber flow cell and exposed to a final concentration of ~10^5^ platelets/μl derived from platelet rich plasma (Bonfils Blood Bank) diluted in PBS supplemented with 5 mM calcium chloride at a physiologically relevant flow rate of 100 s^−1^ for 1 h at 37°C. Surfaces were rinsed in PBS and fixed with 1% paraformaldehyde for 1 h at room temperature. Two 14 mm circular samples were cut from each surface and blocked with 5% goat serum for 1 h prior to staining for fibrinogen with a goat polyclonal primary antibody (ab6666, abcam) for 1 h at room temperature. The primary antibody solution was then exchanged with 5% goat serum, and surfaces were counterstained with a secondary Alexa-Fluor 647 IgG antibody (ab150131, abcam) in 6% goat serum for 1 h. Images were taken in nine pre-determined locations per surface by fluorescent microscopy (Olympus BX3-CBH), area coverage (μm^2^) was measured using ImageJ, and each coverage data point was log transformed prior to statistical analysis. Each experiment was replicated three times.

### Statistical analysis

A log reduction (LR) per experiment was calculated by subtracting the average of the log_10_ transformed values from micropatterned surfaces from the average of the log_10_ transformed values from unpatterned surfaces. After confirming the normality of the log reductions by residual and normal probability plots, the mean log reduction was interpreted as the median percent reduction with the equation: 1-10^(-LR)^. Statistical significance of the reductions were assessed using a 1-sided *t*-test of log reductions compared to zero. Estimates of variances and Tukey tests were assessed using an ANOVA model of the log transformed data for each unpatterned control sample and micropatterned sample, with a random effect for experiment [47]. All analyses were performed using the statistical software MiniTab16.

## Results

### Staphylococcal colonization post blood protein pre-treatment

**Figure 2:**
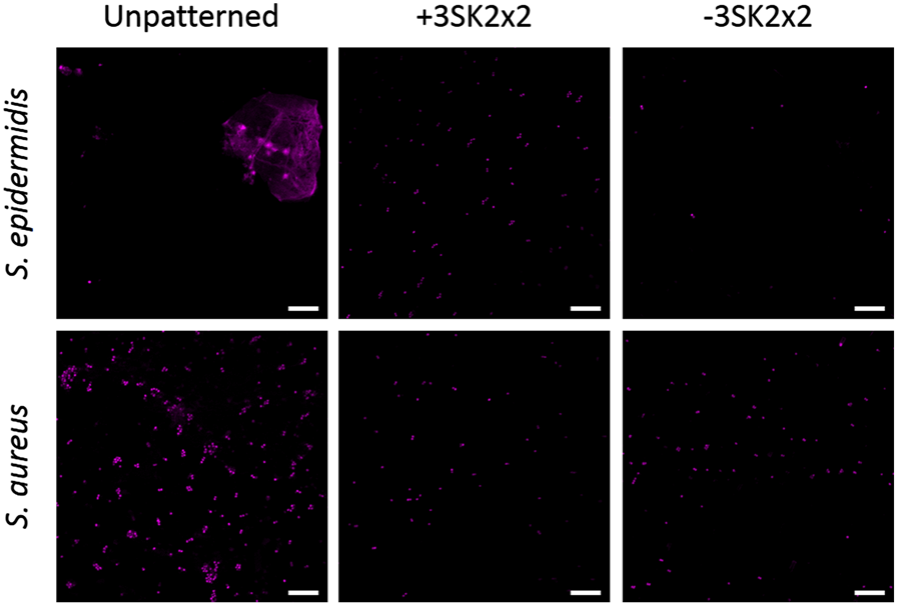
**The Sharklet micropattern reduces the colonization of two common CRBSI-associated pathogens after preconditioning with whole blood.** Representative images of unpatterned, +3SK2x2 and-3SK2x2 TPU surfaces colonized for 18 h with S. epidermidis (top panels) and *S. aureus* (bottom panels). These qualitative images support the quantitative results in Table 1. Scale bar, 10 μm.

In the range of environments selected to model the clinical environment, the-3SK2×2 TPU surfaces demonstrated significant reductions of *S. aureus* colonization ranging from 63% to 70% compared to unpatterned TPU controls, after whole blood pre-conditioning under dynamic and static conditions (Table 1). The ability of the Sharklet micropattern to reduce *S. aureus* colonization was independent of the presence of flow, incubation time and pre-treatment media, based on a Tukey simultaneous test to evaluate significant differences among log reductions (Table 1). *S. epidermidis* attachment to challenge TPU surfaces was below the level of CFU detection when evaluated after whole blood pre-conditioning or under dynamic conditions, similar to previous observations [48]. Thus, 25% serum pre-treatment (a physiologically relevant concentration of blood proteins) with static incubation was the only condition that could be quantitatively evaluated (Table 1).-3SK2×2 TPU surfaces demonstrated reductions of *S. epidermidis* colonization after 18 h by at least 71% (*p* < 0.01) when compared to unpatterned TPU controls under both saline and serum pre-conditioning (Table 1). The capability of the Sharklet micropattern to reduce *S. epidermidis* colonization was independent of conditions, *i.e.* pre-treatment media and inoculation duration, based on a Tukey simultaneous test to evaluate significant differences among log reductions (Table 1). Since quantitative results could not be achieved with *S. epidermidis* after whole blood pre-conditioning, microscopy was conducted to determine whether the Sharklet micropattern would perform in this condition. Micrographs qualitatively demonstrate Sharklet inhibition of *S. aureus* and *S. epidermidis* with whole blood pre-conditioning after 18 h of incubation (Figure 2). An additional Sharklet micropattern (+3SK2×2), in which the features protrude from the surface, was qualitatively assessed for performance as an alternative pattern for a CVC application that may be investigated further in future testing. Both micropatterns demonstrated less microbial colonization and smaller aggregates than control surfaces for the two organisms tested (Figure 2). Large *S. epidermidis* colonies were consistently observed on smooth surfaces (Figure 2).

**Table 1:** **The Sharklet micropattern reduces bacterial colonization after blood protein treatment LD**: ANOVA determination of Average LogCFU/50 mm^2^; SEM: Standard error of the Mean; Conditions that share the same statistical group are not significantly different with *p* > 0.05

**Organism**	**Agitation**	**Incubation period (hr)**	**Pre-condition media**	**Unpatterned LD (SEM)**	**−3SK2x2 LD (SEM)**	**Percent Reduction**	**p value**	**cpi**
S. aureus ATCC 6538	Static	1	Saline	4.05 (0.17)	3.17 (0.20)	94%	0.001	A
								A
			Whole Blood	3.82 (0.13)	3.37 (0.16)	83%	0.003	
		18	Saline	4.91 (0.25)	3.91 (0.21)	89%	0.019	A
			Whole Blood	3.71 (0.29)	3.23 (0.31)	70%	0.048	A
	Dynamic	1	Saline	5.23 (0.22)	4.79 (0.16)	64%	0.128	A
			Whole Blood	4.5 (0.21)	3.9 (0.37)	74%	0.128	A
		18	Saline	5.66 (0.1)	4.82 (0.19)	85%	0.008	A
			Whole Blood	4.49 (0.17)	4.06 (0.23)	63%	0.032	A
S. epidermidis ATCC 35984	Static	1	Saline	5.04 (0.09)	4.23 (0.15)	84%	0.065	A
			25% Serum	3.68 (0.08)	3.24 (0.12)	64%	0.005	A
		18	Saline	5.06 (0.1)	3.92 (0.1)	91%	0.001	A
			25% Serum	3.84 (0.09)	3.47 (0.12)	71%	0.003	A

### Platelet adhesion

**Figure 3:**
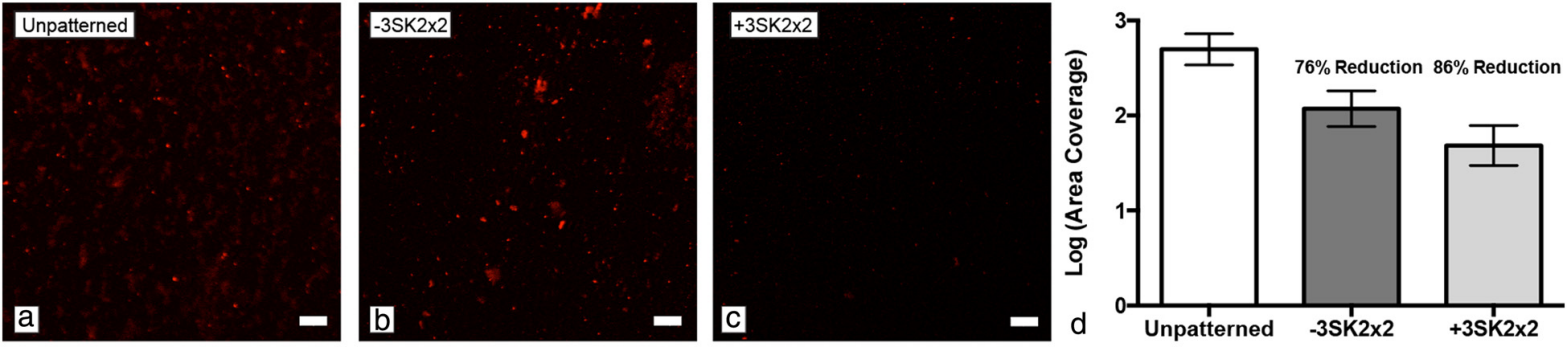
**The Sharklet micropattern reduces platelet adhesion.** Representative images of immunostained platelets on **(a)** unpatterned, **(b)** +3SK2x2 and **(c)** -3SK2x2 TPU surfaces. Quantification of fluorescent images revealed that both Sharklet micropatterns significantly reduce platelet area coverage compared to unpatterned controls. Scale bar, 10 μm.

The first step to reducing the potential for device associated thrombosis is to reduce the level of platelet adhesion to the device. To investigate the potential of the Sharklet micropattern to influence platelet adhesion, −3SK2×2 and +3SK2×2 TPU surfaces were evaluated. Representative images and quantitative analysis show that these surface modifications demonstrated a 76% and 86% (p < 0.05) reduction in platelet area coverage, respectively, when compared to unpatterned TPU controls (Figure 3). The difference in adhesion levels between the two pattern types was not statistically significant (*p*=0.21; Figure 3d).

### Fibrin sheath formation

**Figure 4:**
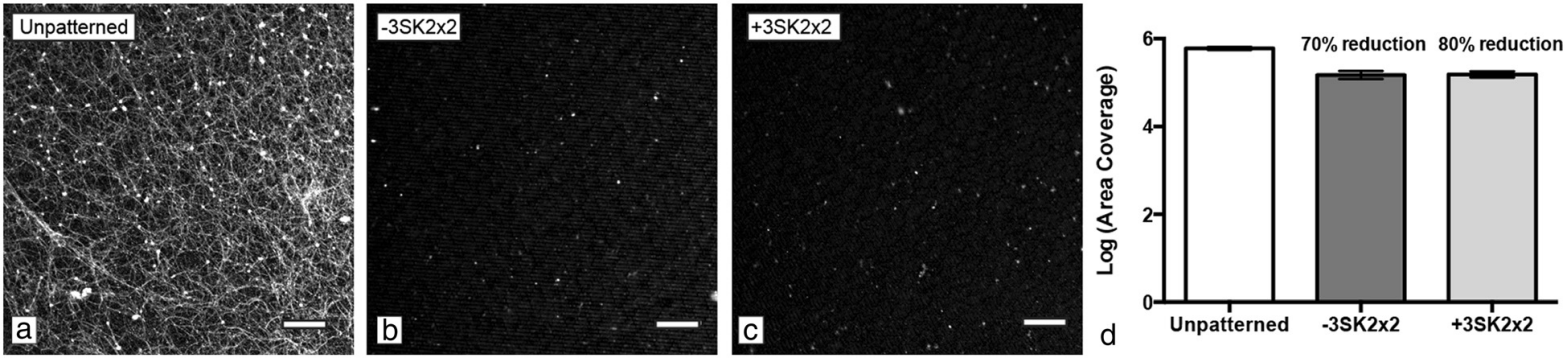
**The Sharklet micropattern reduces fibrin sheath formation resulting from platelet activation.** Representative images of immunostained fibrinogen on **(a)** unpatterned, **(b)** +3SK2x2 and **(c)** -3SK2x2 TPU surfaces. Quantification of fluorescent images revealed that both Sharklet micropatterns significantly reduce fibrinogen area coverage compared to unpatterned controls. Scale bar, 50 μm.

An ideal blood contacting biomaterial would reduce both platelet adhesion and activation to inhibit thrombotic activity. To determine whether the Sharklet micropattern would influence platelet activation in addition to attachment, fibrin sheath formation was measured as a clinically relevant end-point. The-3SK2×2 and +3SK2×2 micropatterns in TPU were evaluated and compared to unpatterned TPU controls. Results show 70% and 80% reductions (p < 0.05) in fibrinogen coverage, respectively (Figure 4). The representative images highlight the differences in fibrin strand formation and coverage on the unpatterned TPU surface compared to the distribution of globular fibrinogen on the micropatterned surfaces (Figure 4). The +3SK2×2 micropattern showed significantly lower fibrin sheath coverage than the-3SK2×2 micropattern across experiments (Tukey test *p *= 0.05, Figure 4d).

## Discussion

Current antimicrobial and heparin coatings used to prevent CRBSI and CRT are limited in their efficacy and are hampered by safety concerns. Micropatterned surfaces have shown promise for reducing bioadhesion in a range of environments and represent a paradigm shift in methods for improving biomaterial compatibility [49-51]. Ordered pillar or well feature configurations have been the primary surface topography evaluated for platelet and bacterial control [30,31,52-58]. However, the Sharklet micropattern has been shown to be the most effective topography for bacterial control with one to three log reductions in microbial attachment across a range of species that cause hospital acquired infections (*i.e. Pseudomonas aeruginosa, Staphylococcus aureus, Escherichia coli, Klebsiella pneumoniae, Acinetobacter baumannii*) [32-34]. The true clinical effect of the Sharklet micropattern is the focus of future investigations based on the promising results of *in vitro* studies such as those presented here. The value of the micropattern approach is the potential to limit bacterial load below a clinically-relevant level without killing bacteria, so as to avoid the emergence of resistant strains of bacteria and difficult-to-treat infections.

The Sharklet micropattern has shown promise for controlling bacterial interaction for several medical device applications (e.g. urinary catheter and endotracheal tubes) [32,33], but has yet to be evaluated for effectiveness in a vascular environment and in a TPU material that is predominately used for vascular access devices. This study is the first of its kind to demonstrate the ability for an ordered micropattern, specifically the Sharklet micropattern, to reduce *S. aureus* colonization after exposure to whole blood (Table 1). This study also presents the first ordered micropattern embossed in TPU to show performance against several CVC fouling agents, *i.e.*, bacterial colonization, platelet adhesion and fibrin sheath formation (Figures 2, 3, 4 and Table 1).

This study demonstrates that not only do fewer platelets adhere to the Sharklet micropattern, but there is also significantly less platelet activation resulting in fibrin sheath formation (Figures 3, 4). Complex fibrin networks are a clinically relevant outcome of platelet adhesion and activation, often leading to CVC occlusion or clots that cause thromboembolisms [14,22]. These fibrin networks have been shown to facilitate microbial colonization and correlate with CRBSIs that occur in the clinic [20,22,24-27]. This study shows that the Sharklet micropattern can individually reduce bacterial colonization in a simulated vascular environment and reduce fibrin sheath formation that can enhance microbial colonization, thereby functioning through a dual approach to potentially limit infections.

Anti-adhesive or anti-biofilm surface alterations may become competitive anti-infective solutions in the near future as the demand increases for alternative approaches [59,60]. Non-wetting topographies, like the Sharklet micropattern, maintain air pockets among features that limit microorganism interaction and provide energetically unstable surfaces for bio-adhesion [37,61]. These surface structures create hydrophobic effects with enhanced liquid pinning/slipping to prevent biofouling [35,62]. For CVC biomaterials, the combined effect of reduced microbial burden (Table 1), smaller microbial aggregates (Figure 2), and reduced fibrin sheath formation (Figure 4) would likely allow for improved immune clearance without the need for multi-log kill effects achieved through antimicrobials.

The Sharklet micropattern with protruding features (+3SK2×2) showed improved performance against platelet adhesion and activation when compared to the micropattern with recessed features (−3SK2×2) (Figures 3, 4). Future work will evaluate feature size and spacing required to achieve optimal boundary layer slip conditions for improved hydrodynamics based on contact line pinning/slipping [63]. It is anticipated that after pattern optimization and manufacturing scale up, a CVC with the Sharklet micropattern will demonstrate reduced CRBSI and CRT rates through preclinical and clinical studies.

## Conclusions

The Sharklet micropattern reduces *S. aureus* and *S. epidermidis* colonization after exposure to a simulated vascular environment by 70% or greater (p < 0.05) when compared to smooth unpatterned controls. This micropattern similarly reduces platelet adhesion and fibrin sheath formation by approximately 80% (p < 0.05). Combined, these results suggest that the Sharklet micropattern implemented onto CVC surfaces will provide clinicians with a non-eluting and non-toxic technology that inhibits the mechanisms that lead to CRBSIs and CRTs.

## Abbreviations

CVC: Central venous catheter
TPU: Thermoplastic polyurethane
CRBSI: Catheter-related blood stream infection
CRT: Catheter-related thrombosis
TSB: Tryptic soy broth
TSA: Tryptic soy agar
PBS: Phosphate buffered saline
CFU: Colony forming unit
LR: Log reduction
